# Predicting Molecular Laser Properties from First-Principles Using Machine Learning-Based Nuclear Ensemble Approach Spectra

**DOI:** 10.1021/acs.jctc.5c01866

**Published:** 2026-04-14

**Authors:** Luis Cerdán, Antonio Francés-Monerris, Michael G. S. Londesborough, Daniel Roca-Sanjuán

**Affiliations:** † Instituto de Química Física Blas Cabrera (IQF-CSIC), 69568 Consejo Superior de Investigaciones Científicas, Madrid 28006, Spain; ‡ Institut de Ciència Molecular, 16781 Universitat de València, P.O. Box 22085, València 46071, Spain; § Institute of Inorganic Chemistry of the Czech Academy of Sciences, Husinec-Řež 250 68, Czech Republic

## Abstract

The accurate prediction of absorption and emission spectra of molecular compounds using quantum mechanical (QM) methods is essential for understanding and designing laser materials, especially when experimental data are limited or inaccessible or when the syntheses are time- and resource-consuming. In this work, a numerical framework is developed to simulate the laser properties of molecular compounds from first principles. The methodology integrates QM calculations in combination with thermal sampling and a Gaussian Mixture Model-based Nuclear Ensemble Approach (GMM-NEA) for spectra reconstruction, and a spectrally resolved spatiotemporal laser simulation model. The GMM-NEA method is extended to include spontaneous and stimulated emission processes, enabling the generation of spectroscopic input for laser modeling. The framework is validated using two boron hydrides, *anti*-B_18_H_22_ and Et_4_-*anti*-B_18_H_18_, which exhibit contrasting laser behaviors, while possessing very similar absorption and emission properties. High-level multireference multiconfigurational QM calculations (CASSCF/MS-CASPT2) are employed, and the results show excellent agreement with experimental data. The present framework identifies excited-state absorption as the primary factor responsible for the absence of lasing in Et_4_-*anti*-B_18_H_18_. This approach represents not only a potentially predictive *in-silico* screening of candidate laser compounds but also offers deeper physical insight into the behavior of novel laser materials.

## Graphical abstract

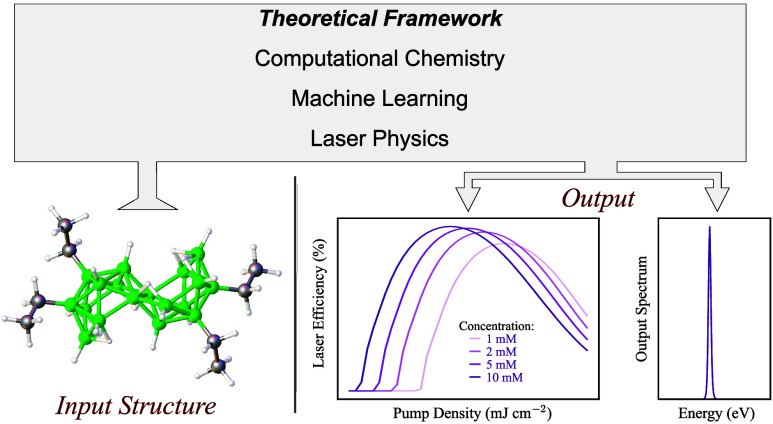

## Introduction

Luminescence, the process by which excited molecules release their energy as spontaneously emitted photons, is at the core of many physical, chemical, and biological processes, and plays a fundamental role in a multitude of applications including microscopy,^1,2^ sensing,^3,4^ and optoelectronics,^5–7^ especially lasers.^8,9^ Among the many molecular materials employed in applications requiring luminescence, the inorganic polyhedral clusters afforded by boron hydrides,^10–12^ or boranes, as they are usually referred to, have received growing attention over the past decade. In two pioneering studies, we first elucidated the distinct photophysical properties of the *anti-* and *syn-*isomers of B_18_H_22_,^13^ and subsequently demonstrated bright and photostable laser emission from the former.^14^ Since then, our groups and others have directed substantial efforts toward the synthesis and characterization of new luminescence boranes.^15–25^ Parallel efforts have explored their potential in other application domains.^16,17,24,26–30^

The polyhedral structure and unique bonding characteristics of boranes lead to an unconventional three-dimensional quasi-aromatic delocalization of the electron density that remains not fully understood.^10^ Thus, computational methods have become essential to explore and understand the energy landscape and structural rearrangements responsible for their photophysical properties.^13,15,16,31,32^ In particular, calculations of the vertical transition energies of absorption or emission at the equilibrium geometries of the ground- and excited-states, respectively, have been instrumental to understand the origin of the thermochromic shift in pyridine substituted boranes,^16^ to delineate the role of upper excited electronic states in the photochemistry and laser performance of *anti-*B_18_H_22_ and its alkylated derivatives,^20,33^ or the prediction of absorption and emission maxima in hypothetical derivatives.^32,34^ However, these single-point calculations offer limited insight into the spectral shape of the transitions, other than assuming a phenomenologically broadened Gaussian line-shape. While useful in certain contexts, this approach lacks the resolution needed to fully capture the complexities of light-induced chemical and physical processes. Moreover, it is well established that the vertical excitation energy calculated for the optimized geometry is usually blue-shifted with respect to the experimentally observed band maximum.^35^

The accurate and reliable prediction of absorption and emission spectra of molecular compounds −not only boranes– by means of quantum mechanical (QM) computations is fundamental for the understanding and discovery of many photophysical, photobiological, photonic, or laser processes in which an experimental determination becomes unfeasible and/or cannot provide sufficient insights into the underlying physics.^36–41^ Among the computational strategies developed for this purpose, the Nuclear Ensemble Approach (NEA) has gained popularity due to its conceptual simplicity and its ability to incorporate nuclear motion effects into electronic spectra.^42,43^ NEA involves generating an ensemble of nuclear geometries around the equilibrium structure −typically sampled from a Wigner distribution– followed by the determination of the vertical excitation energies (Δ*E*) and oscillator strengths (*f*) on top of each geometry. These data are then used to construct the spectrum by assigning a line shape function to each transition. However, a major limitation of traditional NEA implementations is the reliance on phenomenological broadening parameters, such as the full-width at half-maximum (δ), which are often manually selected and lack a rigorous physical basis.

To address this limitation, several authors have employed unsupervised machine learning (ML) techniques to determine the optimal δ for each transition from the available computed Δ*E* and *f*.^44–46^ Recently, we have introduced a ML-based extension of NEA, termed GMM-NEA, which employs Gaussian Mixture Models (GMMs) to reconstruct electronic spectra in a probabilistic data-driven manner.^47^ This approach eliminates the need for arbitrary broadening parameters and has been shown to outperform other data-driven techniques in terms of accuracy and robustness, especially for reconstructions with a small number of geometries. Despite its potential, the current GMM-NEA implementation is restricted to absorption processes, hindering its applicability to the full range of photophysical phenomena, particularly those involving light emission.^48–52^

In this work, an extension of the GMM-NEA methodology to include both spontaneous and stimulated emission processes is incorporated into a comprehensive theoretical framework that enables the simulation of laser properties of molecular compounds from first principles. We first reformulate the NEA equations to account for both spontaneous and stimulated emission electronic spectra in terms of GMM parameters. To simulate the laser dynamics, we integrate the GMM-NEA spectra with a spectrally resolved spatiotemporal rate equations model that accounts for key photophysical processes such as ground- and excited-state absorption, spontaneous and stimulated emission, and both radiative and nonradiative de-excitation pathways. While the reported methodology could be applied to analyze the laser behavior of any luminescent molecular system, we choose boranes as a test bed for three reasons: they are highly promising for light-emitting devices; our strong experimental and theoretical understanding of these compounds allow us to choose the best methodological tools; we have experimental data with which to benchmark the simulations. In particular, we apply this framework to solutions of *anti*-B_18_H_22_ ^14^ and its alkylated derivative Et_4_-*anti*-B_18_H_18_,^20^ two highly emissive compounds with very different laser performances, as shown experimentally in the past. We use an accurate multiconfigurational quantum chemical method to compute the vertical transition energies and oscillator strengths of both ground and excited states for the geometries sampled from Wigner distributions. Our numerical simulations show remarkable agreement with experimental results, both in the GMM-NEA reconstructions of the ground- and excited-state absorption spectra and the stimulated emission cross sections, and in the simulated laser efficiencies and thresholds. By incorporating the uncertainties inherent to the GMM-NEA spectra, we unambiguously confirm that excited-state absorption is the key factor responsible for the lack of laser emission in solutions of Et_4_-*anti*-B_18_H_18_. These results demonstrate the predictive power and versatility of the proposed methodology. By enabling the *in-silico* screening of hypothetical or yet-to-be-synthesized compounds, this approach opens new avenues for the rational design of laser materials and provides a valuable tool for interpreting experimental observations in complex photonic systems.

We must note that previous works have proposed alternative numerical approaches and protocols to perform computational screening of laser materials.^53–57^ However, virtually all studies to date rely on the stimulated emission cross-section at a single wavelength, sometimes along with additional descriptors, as a proxy for the material’s lasing behavior. While this approach has been useful and successful, drawing conclusions solely from single-wavelength parameters without explicitly simulating the laser operation can be misleading, because lasing is a highly nonlinear process governed by many coupled parameters and experimental conditions. As far as we are concerned, our proposed methodology is the first end-to-end approach to predict the actual laser performance from first principles.

## Methodology

### Description of the Proposed Framework

Figure 1 displays a schematic representation of our proposed methodology. It comprises three main components: a first block devoted for quantum mechanical (QM) calculations, including geometry optimizations and vertical transition energies calculations; a second block for the reconstruction of the NEA electronic spectra using probabilistic ML (i.e., GMM-NEA); and a third block for performing laser simulations from the GMM-NEA spectra.

**Figure 1:**
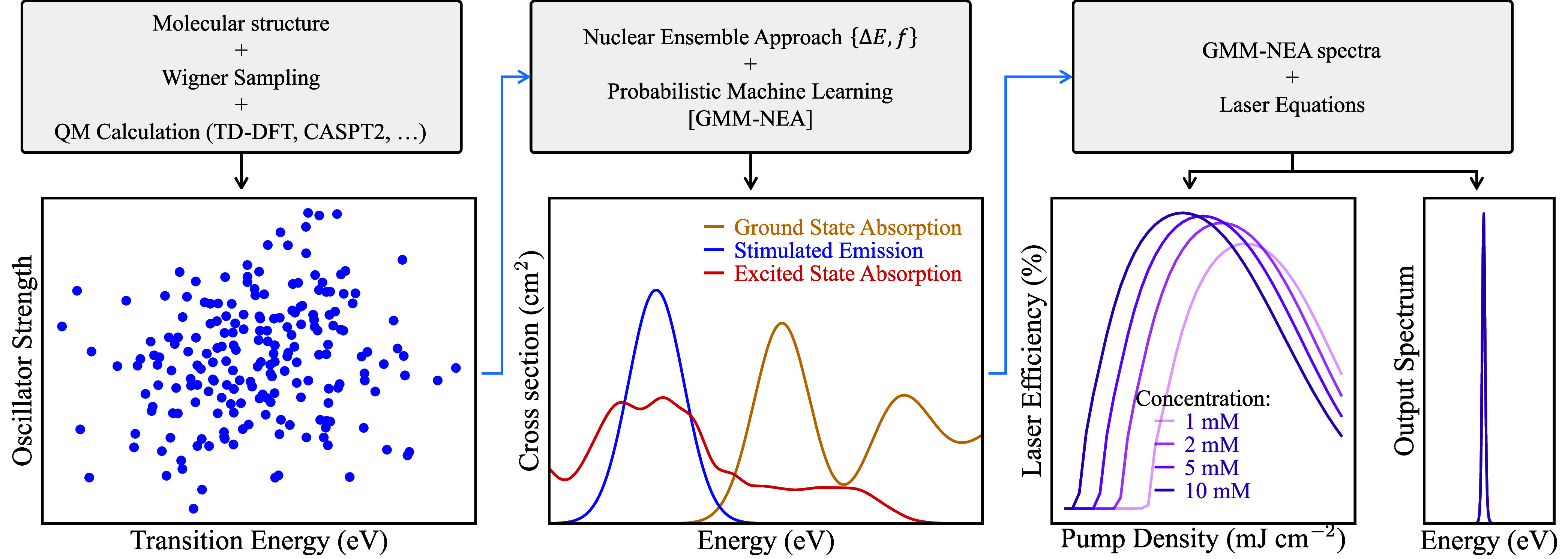
Schematic representation of the proposed theoretical framework to simulate laser properties of a molecular structure from first principles.

QM determinations in the first block initially involve an optimization of both the ground- and excited-state equilibrium geometries, along with their corresponding vibrational harmonic frequencies. In our case, vibrational normal modes were obtained with the density functional theory (DFT)^58^ and its time-dependent (TD-DFT) extension^59^ for the ground- and the excited-state, respectively. Subsequently, a set of geometries around the ground- and excited-state equilibrium structures are sampled from Wigner distributions^60^ using the previously calculated harmonic frequencies.^43^ Finally, a set of Δ*E* and associated oscillator strengths *f* are computed with a multireference method, since they are considered the gold standard for the computation of excited states due to the description of static and dynamic electron correlation.^61,62^ In particular, we resort to the multistate complete active space second-order perturbation theory (MS-CASPT2) approach,^63^ using complete active space self-consistent field (CASSCF) wave functions as reference.^64,65^ The use of multireference methods is particularly important when multiple electron configurations are relevant. The multiconfigurational wave functions built with the state-average CASSCF method offer an inherent advantage as compared to TD-DFT since the latter accounts only for monoelectronic transitions, whereas in the former all possible *n*-electron excitations are considered within the chosen active space. Therefore, it is desirable to use multireference methods to compute high-energy excited states such as those involved in excited-state absorption, where double or even high-order excitations can play a significant role. Further details on the choice of methodologies, and their justification, for the QM calculations are provided below.

As shown in Figure 1, in the second block all pairs of data {Δ*E*, *f*} obtained in the first step are used to generate the ground- and excited-state spectra (absorption and emission) using GMM-NEA to reconstruct the NEA electronic spectra in a probabilistic, data-driven manner. In ref 47, and in the Supporting Information of the current manuscript, we show that the key of GMM-NEA is to first convert oscillator strength *f* to the modulus of the dipole moment *M* (to avoid skewness in their values and improve the GMM fit), and then estimating the probability density function P(ΔE,M) underlying the available pairs {Δ*E*, *M*}. This can be done by means of GMMs, a sum or mixture of *K* bivariate normal distributions, as $P\left(\Delta E,M\right)=\sum_{k=1}^{K}\pi_{k}\phi \left(\Delta E,M;\mu_{k},\Sigma_{k}\right)$ , where π_*k*_ are the weights of each normal distribution, and ϕ­(Δ*E*, *M*; **μ**_*k*_, **Σ**_*k*_) are bivariate normal distributions with vector of means **μ**_*k*_ = (μ_*k*,1_, μ_*k*,2_) and covariance matrix **Σ**_*k*_ = (σ_*k*,1_^2^, ρ_*k*_σ_*k*,1_σ_*k*,2_; ρ_*k*_σ_*k*,1_σ_*k*,2_, σ_*k*,2_^2^), with σ_*k*,1_^2^ and σ_*k*,2_^2^ the variances of the mixture covariates and ρ_*k*_ their correlation coefficient. The subscripts 1 and 2 refer, respectively, to the corresponding variable Δ*E* and *M*. By exploiting the properties of the normal distribution, one can express the NEA equation for the cross-section in terms of GMM parameters instead of Δ*E* and *M* and, at the same time, eliminate the need of the empirical broadening parameter δ. In our previous work, we employed this strategy to derive the GMM-NEA expression for the absorption cross-section spectrum of an individual electronic transition *n* in a molecular system, which reads:

1 $$\sigma_{\mathrm{abs},n}\left(E\right)=\frac{\pi F\left(n_{r}\right)E}{3\hbar c\epsilon_{0}n_{r}}\sum_{k=1}^{K_{n}}\pi_{n,k}\left({\mathrm{\mu ̃}}_{n,k}^{2}+{\mathrm{\sigma ̃}}_{n,k}^{2}\right)\phi \left(E;\mu_{1,n,k},\sigma_{1,n,k}^{2}\right)$$

where *E* is the photon energy, *c* is the speed of light in vacuum, ℏ is the reduced Planck constant, and ϵ_0_ is the vacuum permittivity. The factor *F*(*n*_*r*_) = (3*n*_*r*_^2^/(2*n*_*r*_^2^+1))^2^ accounts for the refractive index *n*_*r*_ induced modification of the transition rate, following the real-cavity approximation.^66^ Moreover, ϕ­(*E*; μ_1,*n*,*k*_, σ_1,*n*,*k*_^2^) are normal distributions, while the parameters μ̃_*n,k*_ and σ̃_*n,k*_^2^ are defined as

2 $${\mathrm{\mu ̃}}_{n,k}=\mu_{2,n,k}+\rho_{n,k}\frac{\sigma_{2,n,k}}{\sigma_{1,n,k}}\left(E-\mu_{1,n,k}\right)$$

3 $${\mathrm{\sigma ̃}}_{n,k}^{2}=\left(1-\rho_{n,k}^{2}\right)\sigma_{2,n,k}^{2}$$

The parameters *K*, π_*n,k*_, μ_1,*n,k*_, μ_2,*n,k*_, σ_1,*n,k*_^2^, σ_2,*n,k*_^2^, and ρ_*k*_ are estimated during the fitting of the GMM to the available pairs {Δ*E*, *M*}. The final GMM-NEA absorption cross-section is constructed as the incoherent sum of the contributions from all possible excited-states *N*_*s*_ as σ_abs_(*E*) = ∑_*n* = 1_^*N*_*s*_^σ_abs,*n*_(*E*). This formulation is valid for both ground-state and excited-state absorption.

In this manuscript, we derive for the first time the GMM-NEA expressions for the spontaneous emission spectrum, expressed as the differential radiative decay rate Γ_r_(*E*),^43^ and for the stimulated emission cross-section spectrum σ_se_(*E*). As detailed in the Supporting Information, these are given respectively by

4 $$\Gamma_{r}\left(E\right)=\frac{n_{r}F\left(n_{r}\right)E^{3}}{3\pi \hbar^{3}c^{3}\epsilon_{0}}\sum_{k=1}^{K}\pi_{k}\left({\mathrm{\mu ̃}}_{k}^{2}+{\mathrm{\sigma ̃}}_{k}^{2}\right)\phi \left(E;\mu_{1,k},\sigma_{1,k}^{2}\right)$$

5 $$\sigma_{\mathrm{se}}\left(E\right)=\frac{\pi F\left(n_{r}\right)E}{3\hbar c\epsilon_{0}n_{r}}\sum_{k=1}^{K}\pi_{k}\left({\mathrm{\mu ̃}}_{k}^{2}+{\mathrm{\sigma ̃}}_{k}^{2}\right)\phi \left(E;\mu_{1,k},\sigma_{1,k}^{2}\right)$$

where we assume for simplicity that only one electronic excited state contributes to the emission spectra. As a result, μ̃_*k*_ and σ̃_*k*_^2^ are given by eqs 2 and 3, respectively, but without the index *n*, since only a single transition is considered. The radiative decay rate is obtained by direct integration as^43^

$$\gamma_{r}=\frac{1}{\hbar }\int \Gamma_{r}\left(E\right)dE$$ 6

Figure 2 shows a visual explanation of the procedure followed to obtain the GMM-NEA spectrum from a set of NEA calculations. At a very high level, the process involves: (i) identifying the optimal GMM hyperparameters (number of mixtures *K* and covariance-matrix structures), that ensure model generalization and accuracy, using the set of {Δ*E*, *M*} pairs with a nonzero value in *M* (Figure 2a); (ii) extracting the estimated means, variances, correlation coefficients, and weights associated with each mixture component of the optimized GMM; and (iii) substituting these parameters into eqs 2 and 3 to reconstruct the electronic spectrum using eqs 1, 4, or 5, depending on the desired output. Each mixture’s mean and confidence ellipse in Figure 2a define, respectively, the peak position and width of the individual contributions to the total spectrum shown in Figure 2b. Additional details on the procedure, as well as on the GMM model selection and optimization, can be found in the Supporting Information. Implementation details of our GMM-NEA approach are provided below.

**Figure 2:**
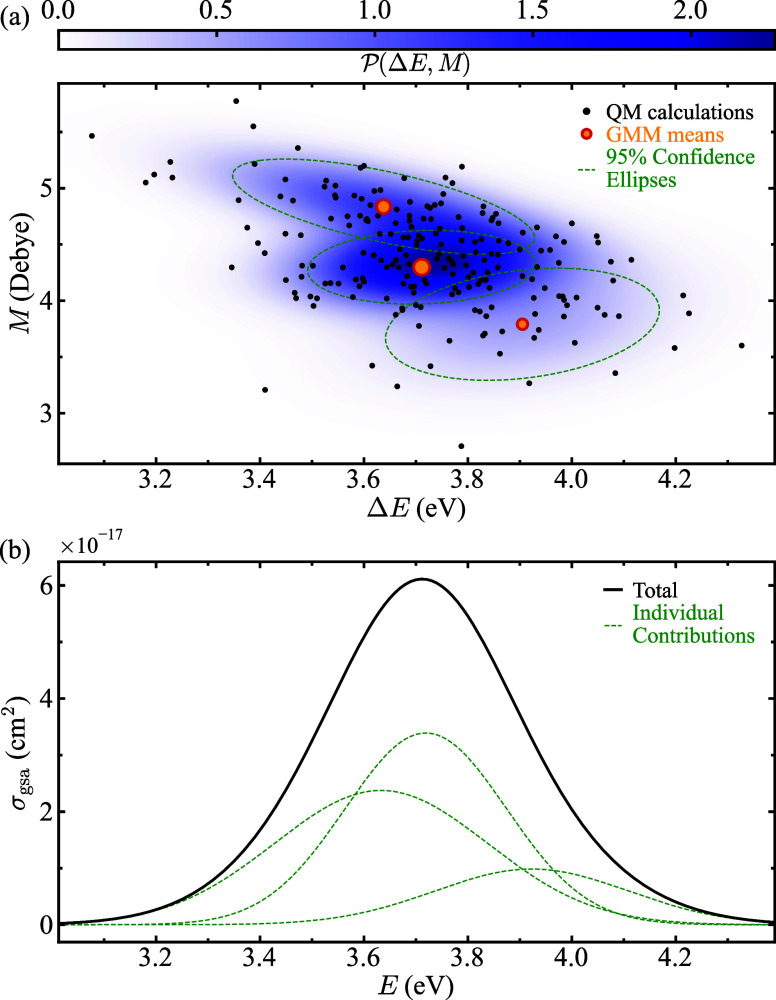
(a) Example of {Δ*E*, *M*} data pairs obtained for NEA QM calculations (black points), along with the joint probability density function P(ΔE,M) fitted using a GMM with three components (*K* = 3, blue colormap). The yellow points and green ellipses represent the means and 95% confidence ellipses of each mixture, respectively. Note that with small or anisotropic samples, points outside the 95% confidence ellipses are expected due to sampling variability and do not signal model misfit. (b) GMM-NEA spectrum (black solid line) obtained from the data in (a), with the individual contributions from each component shown as green dashed lines.

In the third block (cf. Figure 1), the results from the GMM-NEA step are used to provide the spectroscopic input for a system of equations that simulate the laser properties. The laser system under consideration is schematically depicted in Figure 3a. We consider a plane-parallel cavity aligned along the *x*-axis, formed by a back mirror with reflectivity *R*_1_, and a quartz cuvette that serves both as the container for the isotropically oriented laser solution and as an output coupler, with a Fresnel reflectivity *R*_2_. The laser solution is transversely pumped at a wavelength λ_p_ by a laser beam with a Gaussian temporal profile, spatially conformed as a rectangular stripe, and propagating along the *y*-axis. The excited region has length *L*, width *w* (*L* ≫ *w*), and depth *d* (*d* ∼ *w*), determined by the pump focusing and penetration depth, respectively. The latter depends on the doping volume concentration *N*_D_ and the ground-state absorption cross-section σ_gsa,p_ (evaluated at λ_p_) as *d* = 1/(σ_gsa,p_ *N*_D_). The pump polarization is perpendicular to the plane defined by the excitation and output beams, resulting in emitted light that is predominantly polarized along the *z*-axis. While the actual polarization would depend on the relative orientation of the absorption and emission transition dipole moments,^67^ assuming a linearly polarized output emission under nanosecond excitation is a reasonable approximation and, in fact, it would not affect the output laser efficiency, as shown experimentally before.^68^ Under these conditions, two counter-propagating waves are excited along the *x*-axis (Figure 3a): the forward *I* ^+^(*x*, *t*, λ) and backward *I* ^–^(*x*, *t*, λ) propagating photon fluxes.

**Figure 3:**
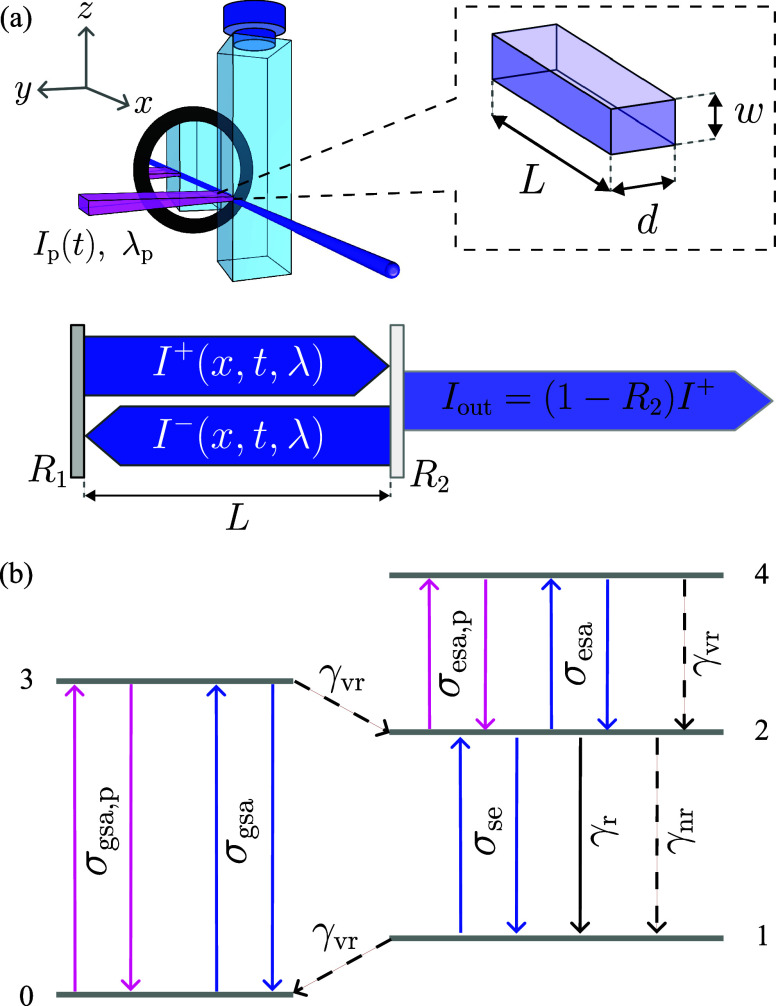
(a) Schematic of the laser system configuration under consideration, consisting of a plane-parallel cavity formed by a back mirror of reflectivity *R*_1_ and a quartz cuvette, with reflectivity *R*_2_, which contains the active medium. The system is transversely pumped by a Gaussian pulse *I*_p_(*t*) at a wavelength λ_p_. Under these conditions, both forward *I* ^+^(*x*, *t*, λ) and backward *I* ^–^(*x*, *t*, λ) photon fluxes propagate within the cavity. The zoom-in region illustrates the geometry of the excitation region. (b) Jablonski diagram showing the electronic levels and GMM-NEA transitions considered for the laser simulation. γ_r_, γ_nr_, and γ_vr_ represent the radiative, nonradiative, and vibrational relaxation rates, respectively.

Figure 3b displays the Jablonski diagram summarizing the photophysical processes that contribute to the laser action and population dynamics. This diagram is expressed in terms of GMM-NEA derived cross sections. We consider a five-level system, with states labeled 0 to 4. In order to avoid confusion with the QM terminology, a note is needed on the notation used for the laser simulations. Level 0 represents the ground state S_0_ at the equilibrium geometry. Level 3 (4) acts as a “metastate” accounting for all the excited states S_*i*_ accessible from S_0_ (S_1_). Level 2 represents the first, lowest lying, excited state S_1_ at the equilibrium geometry. Finally, level 1 represents as well the ground state S_0_, but at a nonequilibrium geometry. Upon absorption of a pump photon, a molecule is promoted from the ground state 0 to the excited level 3, and subsequently relaxes nonradiatively to the metastable laser level 2 via vibrational relaxation, or returns to 0 via stimulated emission. From the laser level 2, the molecule can decay to 1 either radiatively via spontaneous or stimulated emission, or nonradiatively via thermal dissipation. Alternatively, it can promote to 4 through absorption of a pump or cavity photon, followed by relaxation back to the initial state via vibrational relaxation or stimulated emission. Once in 1, the molecule can relax to the ground state 0 through vibrational relaxation or be re-excited to 2 by absorbing a cavity photon. The probabilities of all the radiative and absorptive processes are governed by the cross sections and radiative decay rates obtained from the GMM-NEA analysis. Although the nonradiative decay rates γ_vr_ and γ_nr_ can be computed numerically,^69–71^ for the purpose of this study we use fixed values for them, as described in the Results section. In addition, in ref 33 we showed for *anti*-B_18_H_22_ that once it was promoted to an upper excited state (level 4 in the Jablonski’s diagram), it could undergo a photofragmentation pathway leading to the irreversible quenching of the emitter fluorescence. This deactivation pathway has not been included in the laser simulations since it is an effect that becomes relevant only for intense and prolonged laser excitation, a situation that is avoided in this work, where single pulse experiments on fresh samples are assumed.

Given the laser material and configuration selected for this study, we employed a rate equations model^72^ to simulate the laser properties (see details in the Supporting Information). This methodology enables a fully spectrally resolved, spatiotemporal description of the laser flux and populations within the cavity, and yields reliable results as long as the phase of the electric field and matter polarizations is not critical.^8^ As the output of the simulation is spectrally resolved, we can obtain the peak wavelength, where the maximum photon flux is extracted, or the full width at half-maximum, which provides information on the amplification capabilities. The time-domain dependency can be exploited to gain insights on the dynamics of the level populations and laser emission, especially for pulsed-operation. Of particular relevance to our study is the simulation of the laser efficiency, η, defined as the ratio between the output energy *E*_out_ and the input pump energy *E*_p_. Within our framework, this quantity is obtained by integrating the photon flux exiting the cavity over time and wavelengths, as

7 $$\eta =100\cdot \left(1-R_{2}\right)\frac{\mathrm{wd}}{E_{p}}\iint_{-\infty }^{+\infty }I^{+}\left(L,t,\lambda \right)\frac{\mathrm{hc}}{\lambda }d\lambda dt$$

Finally, by plotting the laser efficiency η, or *E*_out_, as a function of the pump energy *E*_p_ and identifying the point where the slope suddenly changes, we can obtain the laser threshold, the energy at which lasing begins. In this manuscript, we will mainly focus on the laser efficiency, with some discussion on the laser threshold. We performed several tests to verify that our implementation was functioning correctly and producing reliable results.

### Computational Details

All QM calculations were performed in vacuum without imposing any symmetry constraint. The ground-state geometry optimizations and harmonic vibrational frequencies for *anti*-B_18_H_22_ and Et_4_-*anti*-B_18_H_18_ were calculated using DFT with the B3LYP functional and the 6-31G*basis set. For excited-state optimizations and frequencies, we employed TD-DFT with the same functional and the 6-31+G­(d) basis set. The choice of this functional and basis set entirely relies on our previous experience in describing the molecular properties of boron hydrides and related structures, repeatedly validated in the literature by comparison with experimental NMR and UV/vis spectra, and crystallographic structures.^13,17,20,32,73^ All DFT and TD-DFT computations were carried out using the Gaussian 09 software package.^74^ NEA geometries sampled from a Wigner distribution were generated with the Newton X program, version 1.4.^75^ Vertical absorption energies and oscillator strengths for a total of 10 singlet electronic states were determined on top of the 200 sampled ground-state geometries using the state-averaged (SA)-CASSCF/MS-CASPT2 methodology. SA-CASSCF is used to build multiconfigurational wave functions and MS-CASPT2 is used to compute the necessary electronic dynamic correlation. The MS variant of CASPT2 is the most appropriate choice for the calculation of vertical excitation properties near Franck–Condon points and outside state-crossing regions.^76,77^ Excited-state absorption and emission computations were carried out on top of the 200 excited-state geometries sampled around the S_1_ minimum, employing the same methodology, considering 20 electronic states to ensure coverage of all relevant spectroscopic wavelengths. Wave functions were built by distributing 12 electrons into 12 molecular orbitals, an active space that has been shown to be adequate through validation against experimental data in previous studies on the same^13,20,33^ and related systems.^15,17,32^ While the agreement between theory and experiment is not perfect, partly due to active space truncation among other factors (see Results section), the discrepancies are not large enough to affect the conclusions of our study. The interested reader can find the orbitals of the active space used for *anti*-B_18_H_22_ and Et_4_-*anti*-B_18_H_18_ in Figure S1 of the Supporting Information. In MS-CASPT2 calculations, an imaginary level shift of 0.2 au was applied to mitigate the influence of weakly interacting intruder states, whereas the ionization-potential electron-affinity (IPEA) shift was set to 0.25 au, consistent with previous studies.^13,15,17,32,33^ All multiconfigurational calculations were performed with the ANO-S-VDZP double-ζ basis set as implemented in the OpenMolcas software package.^78,79^ All these QM calculations were managed using the MULTISPEC wrapper, available at https://github.com/qcexval/multispec.

The methods for calculating the GMM-NEA spectra have been implemented in R. Specifically, we used the *mclust* package (version 5),^80^ a powerful and versatile tool for modeling data with GMMs using the Expectation-Maximization algorithm for classification, clustering and density estimation. This package supports automatic model selection via maximization of the Bayesian Information Criterion, considering a range of covariance structures (model constraints M ) and different numbers of mixture components *K*. A fully functional and flexible version of the code for computing GMM-NEA ground-state absorption, excited-state absorption, stimulated emission, transient absorption, and the differential decay rate is available in the Git-Hub repository https://github.com/lucerlab/GMM-NEA under a LGPL-2.1 License.

## Results

We tested the validity of the proposed framework and methodologies on two different boron hydrides: *anti*-B_18_H_22_ and its alkylated derivative Et_4_-*anti*-B_18_H_18_. Both compounds are air-stable crystalline solids, amenable and safe for handling and manipulation, but readily soluble in organic solvents, where the experiments are usually conducted. The molecular structures of both species, displayed in Figure 4a and d, comprise the same octadecaborane cluster structure, with the only difference being the substitution of hydrogen atoms for ethyl (−CH_2_CH_3_) groups at four of the boron-cluster positions. This alkyl-substitution resulted in the increased solubility of the compound in nonpolar organic solvents used in photophysical and laser studies.^20^ As shown in Figure 4b and e, solutions of both compounds exhibit well-resolved, broad absorption and emission bands, and display very similar ground-state absorption (blue lines) and stimulated emission (yellow lines) cross sections among them. Despite these similarities, *anti*-B_18_H_22_ exhibits laser emission with measured efficiencies of up to 10%,^14^ whereas Et_4_-*anti*-B_18_H_18_ does not lase, even though it has a unit photoluminescence quantum yield.^20^ In the latter study we showed, using an ultrafast transient UV/vis absorption spectroscopic study of the tetraethylated compound, an efficient excited-state absorption at the emission wavelength. We hypothesized that it would inhibit its laser emission, although it was not explicitly proven. The application of our framework to these systems offers a means to test and potentially confirm this hypothesis.

**Figure 4:**
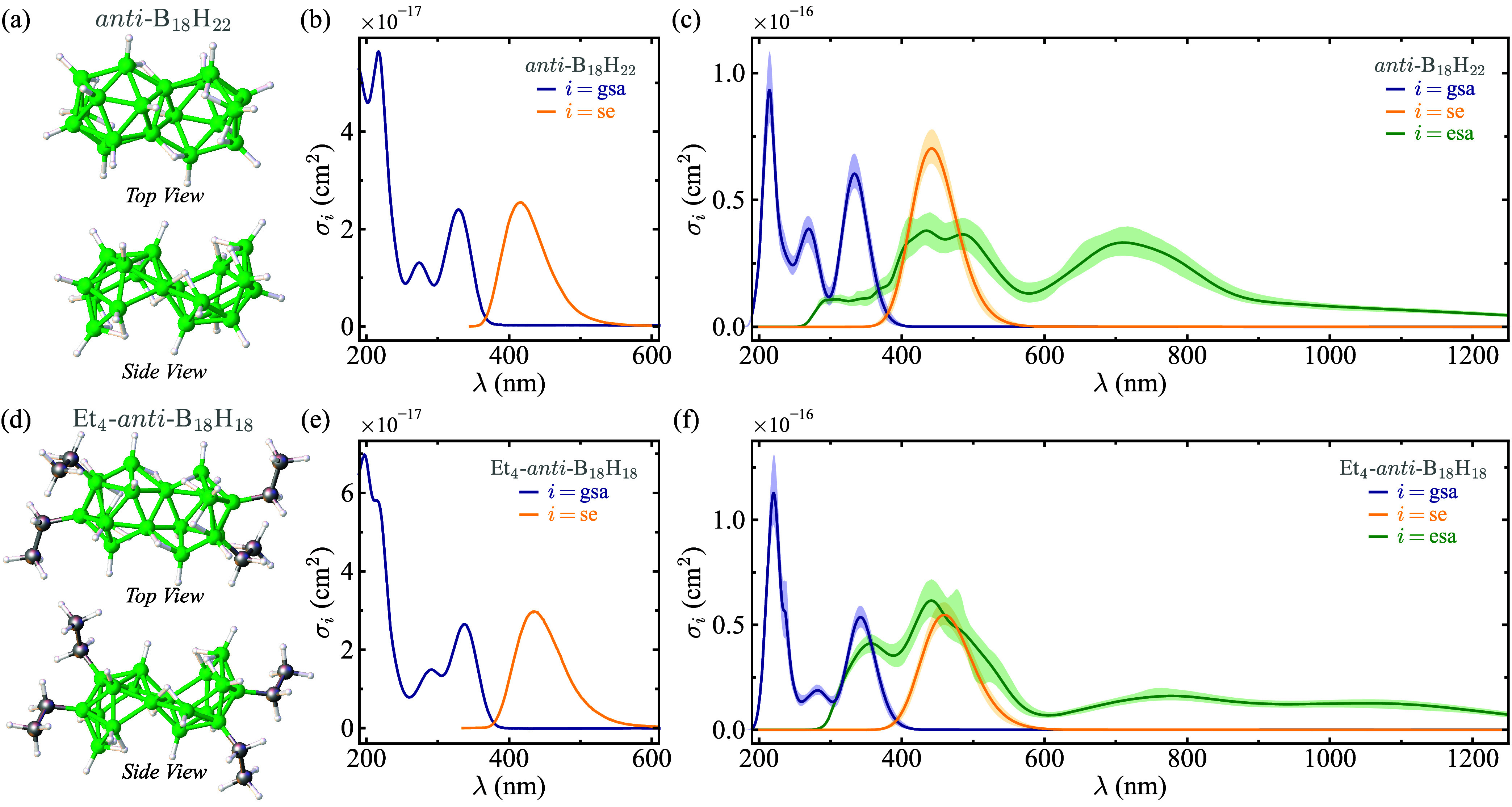
(a) Ground-state equilibrium geometry of *anti*-B_18_H_22_, optimized using DFT. (b) Experimental cross sections for ground-state absorption (gsa, blue line) and stimulated emission (se, yellow line) of *anti*-B_18_H_22_ in cyclohexane solution. (c) GMM-NEA cross sections for ground-state absorption (blue line), stimulated emission (yellow line), and excited-state absorption (esa, green line) of *anti*-B_18_H_22_. Shaded areas represent 95% confidence intervals. (d), (e), and (f) are analogous to (a), (b), and (c), respectively, but for Et_4_-*anti*-B_18_H_18_. The experimental σ_se_ spectra are computed from the measured steady-state spontaneous emission spectra *F* with the Füchtbauer-Ladenburg equation σ_se_ = λ^4^ *F*/8*πcn*_*r*_^2^τ, where τ is the measured fluorescence lifetime and *F* is normalized as ∫*Fdλ* = ϕ, with ϕ the measured photoluminescence quantum yield.^81^ For the GMM-NEA calculations, we used 200 geometries and assumed a refractive index of *n*_*r*_ = 1.38 (cyclohexane) for the three spectral bands.

In the context of NEA, increasing the number of sampled geometries improves the spectral reconstruction but also entails longer computation times −especially when using high-level QM methods or dealing with complex systems. In our previous work,^47^ we introduced the sequential band-wise relative integral change (*bRIC*_seq_), a metric designed to quantify the number of geometries required to achieve sufficiently converged GMM-NEA spectra. This metric measures how much the reconstructed spectrum changes upon the addition of a new batch of samples; accordingly, *bRIC*_seq_ tends toward zero as the number of batches increases. Figure S2a and b in the Supporting Information show the *bRIC*_seq_ for the ground-state, excited-state, and stimulated emission spectra of *anti*-B_18_H_22_ and Et_4_-*anti*-B_18_H_18_, respectively. For 200 geometries, *bRIC*_seq_ falls to or below 0.05 (within our uncertainty bounds) both for the ground- and excited-state spectra. This threshold was identified as an indicative of sufficiently converged GMM-NEA spectra, since any further addition of geometries led only to slight visual spectral changes.

Figure 4c and f display the GMM-NEA cross sections for ground-state absorption (blue lines), stimulated emission (yellow lines), and excited-state absorption (green lines) for *anti*-B_18_H_22_ and Et_4_-*anti*-B_18_H_18_, respectively, using 200 geometries in each case. Notice that the QM calculations were performed in vacuum, principally because solvent treatment approaches are quite challenging if used together with multiconfigurational quantum chemistry. Accordingly, solvent effects were included only through the refractive index term *F*(*n*_*r*_) in eqs 1, 4, or 5. The GMM-NEA spectra exhibit remarkable agreement with the experimental spectra shown in Figure 4b and e, both in terms of band shapes and positions, as well as relative peak cross sections. For *anti*-B_18_H_22_, the GMM-NEA excited-state absorption cross-section spectrum reveals prominent bands that overlap with both the main ground-state absorption and stimulated emission bands. These results confirm the findings of our previous single-geometry calculations,^33^ which identified the excited-state absorption as a key factor contributing to the photochemical degradation and reduced laser efficiency in this compound. The excited-state absorption spectrum in Figure 4f suggests that this effect is even more pronounced in Et_4_-*anti*-B_18_H_18_, supporting the hypothesis that the lack of lasing in this derivative is due to excessive excited-state absorption.

From an experimental standpoint, retrieving the excited-state absorption cross-section is challenging, and measuring the differential transient absorption is generally preferred. In pump–probe experiments, the transient absorption is defined as Δ*A* = *A* ^off^ – *A* ^on^, i.e., the difference between the probe absorbance in the absence (*A* ^off^) and presence (*A* ^on^) of a pump excitation pulse. Figure 5a shows the experimentally measured Δ*A* at a probe delay of 15 ps – sufficient time for the molecules to relax to their lowest-laying excited state– for *anti*-B_18_H_22_ (blue line) and Et_4_-*anti*-B_18_H_18_ (yellow line). Under these conditions, Δ*A* is proportional to σ_esa_–σ_gsa_–σ_se_,^82^ that can be understood as the transient absorption cross-section σ_ta_. This quantity is plotted in Figure 5b for *anti*-B_18_H_22_ (blue line) and Et_4_-*anti*-B_18_H_18_ (yellow line), as calculated from the GMM-NEA spectra in Figure 4c and f. Once again, a good qualitative agreement is observed between the experimental results and the GMM-NEA reconstructions. The spectra in Figure 5 evidence that *anti*-B_18_H_22_ exhibits a region of negative absorption (i.e., optical gain) near its emission band (cf. Figure 4c and d), along with a strong positive absorption feature −indicative of excited-state absorption– centered around 520 nm. In contrast, Et_4_-*anti*-B_18_H_18_ displays no gain in the experimental data and only a negligible gain near 470 nm in the GMM-NEA reconstruction. However, when considering the confidence intervals, it is likely that this compound does not exhibit any gain at all. This lack of gain was the key feature that led to the hypothesis that excited-state absorption was responsible for the absence of lasing in this derivative.^20^

**Figure 5:**
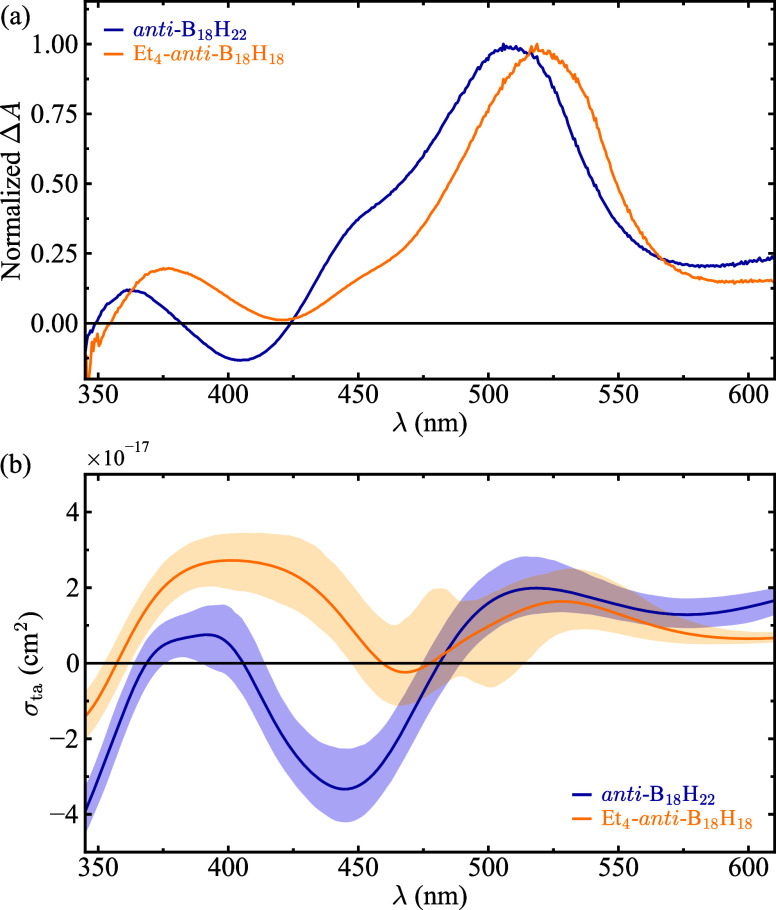
(a) Experimental differential transient absorption (Δ*A*) spectra for *anti*-B_18_H_22_ (blue line) and Et_4_-*anti*-B_18_H_18_ (yellow line) in hexane solution, obtained at a pump excitation wavelength of 340 nm and a probe delay of 15 ps. See ref 20 for experimental details. No copyright permissions are required. (b) GMM-NEA transient absorption cross-section for *anti*-B_18_H_22_ (blue line) and Et_4_-*anti*-B_18_H_18_ (yellow line). Shaded areas represent 95% confidence intervals.

To confirm this hypothesis, we simulated the laser performance of solutions of both compounds using the GMM-NEA spectra shown in Figure 4, along with the radiative decay rates computed using eq 6. For these simulations, we assumed the cavity configuration depicted in Figure 3a, with *L* = 1 cm, *w* = 200 μm, refractive index *n*_*r*_ = 1.38 (cyclohexane), and mirror reflectivities *R*_1_ = 0.9 and *R*_2_ ≈ 0.04. The solutions were excited by Gaussian pulses of full-width half-maximum duration τ_p_ = 4 ns and wavelength 337 nm. These parameters are consistent with previous experimental results.^14^ Additionally, we assumed a vibrational relaxation rate of γ_vr_ = 1 ps and photoluminescence quantum yields ϕ = γ_r_/(γ_r_ + γ_nr_) = 1 for both compounds. A discussion of the effect of these parameters is provided below. Figure 6a shows the computed laser efficiency and threshold for solutions of both compounds as a function pump density (defined as the energy *E*_p_ over the excitation area *wL*), evaluated at several compound concentrations. These curves exhibit the typical laser behavior expected for molecular systems.^67^ At low pump densities, lasing does not occur because the population inversion is insufficient for the gain to overcome material and cavity losses, modeled in the laser simulations through the ground- and excited-sate reabsorption and the nonunity reflectivity of the feedback mirrors, respectively. Once the pump density exceeds the threshold, the gain surpasses the losses, and the solution begins to emit laser light, albeit with low efficiency. As the pump density increases further, the laser efficiency initially rises sharply, then saturates, and eventually diminishes. This decline results from a combination of pump saturation (where the ground state becomes transparent to the pump radiation), gain saturation (where the laser transition becomes transparent to the cavity photons), and increasing excited-state absorption, which traps the molecules in nonlasing states (specifically, the 2 ↔ 4 transition shown in Figure 3b). Furthermore, increasing the concentration significant reduces the laser threshold and moderately increases the maximum laser efficiency.

**Figure 6:**
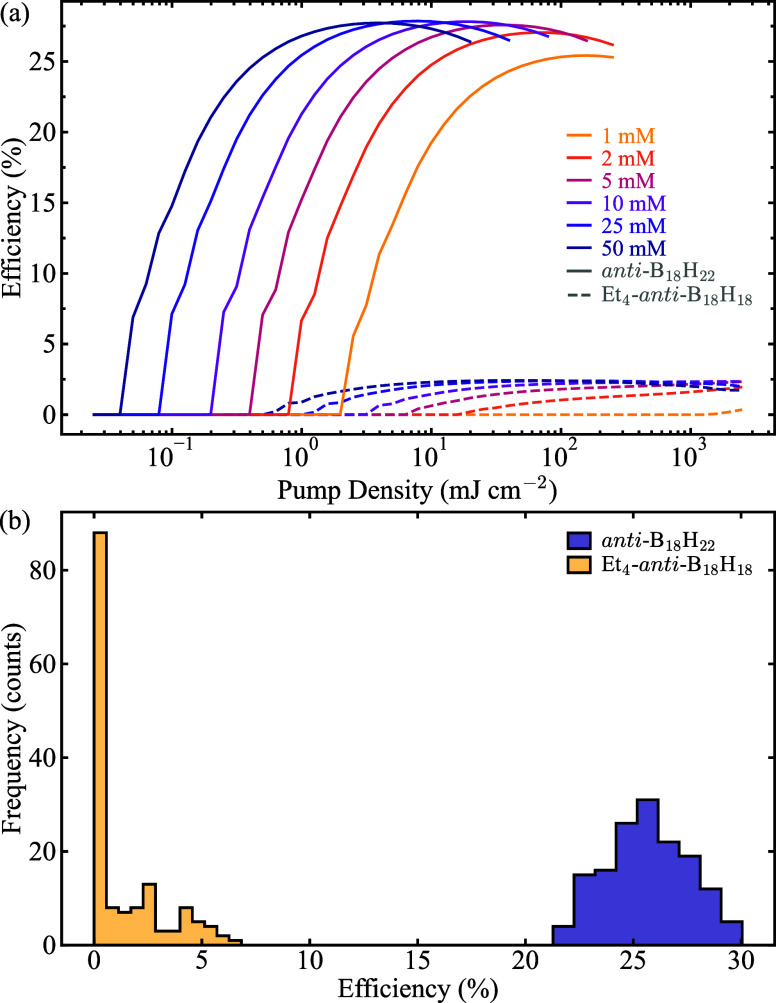
(a) Simulated laser efficiency of solutions of *anti*-B_18_H_22_ (solid lines) and Et_4_-*anti*-B_18_H_18_ (dashed lines) as a function of pump density, for increasing compounds concentration. (b) Distribution of laser efficiency for *anti*-B_18_H_22_ (blue shaded histogram) and Et_4_-*anti*-B_18_H_18_ (yellow shaded histogram), built on 150 independent random samplings of the effective spectra σ_*i*_^eff^ (see text for details), for 10 mM solutions excited at a pump density of 50 mJ cm^–2^.

Nevertheless, the two boranes studied here exhibit markedly different behavior in terms of laser efficiency and threshold. While solutions of *anti*-B_18_H_22_ display moderate efficiencies up to ∼ 27% (solid lines in Figure 6a), the solutions of Et_4_-*anti*-B_18_H_18_ reaches at most efficiencies ∼ 3%, with laser thresholds an order of magnitude higher (dashed lines in Figure 6a). These results align with the experimental observations, which show that *anti*-B_18_H_22_ ^14^ lases with a moderate efficiency, whereas Et_4_-*anti*-B_18_H_18_ does not.^20^ Still, the simulations suggest that Et_4_-*anti*-B_18_H_18_ should emit laser with low but measurable efficiency, likely due to the negligible gain near 470 nm observed in Figure 5b. However, when considering the reconstruction confidence intervals, this weak laser signal is expected to vanish. To account for the confidence intervals of the GMM-NEA spectra in the laser simulations, we performed a Monte Carlo experiment. Instead of using the spectra directly as calculated from eqs 1 and 5 (solid lines in Figure 4c and f), we used the effective spectra defined as σ_*i*_^eff^ = σ_*i*_ + φ_*i*_|Δ*σ*_*i*_| (*i* = gsa, esa, se), where φ_*i*_ are random values sampled from independent uniform distributions between −1 and 1, and Δ*σ*_*i*_ represent the upper or lower confidence intervals. If φ_*i*_ < 0, Δ*σ*_*i*_ corresponded to the lower confidence interval; otherwise, the upper one was used. Using these effective spectra, we simulated the laser properties. Figure 6b displays the resulting distribution of simulated laser efficiencies for *anti*-B_18_H_22_ (blue shaded histogram) and Et_4_-*anti*-B_18_H_18_ (yellow shaded histogram), at a concentration of 10 mM and a pump density of 50 mJ cm^–2^, based on 150 independent random samplings of φ_*i*_. While *anti*-B_18_H_22_ maintains a consistent laser efficiency with a narrow dispersion (mean 26%; standard deviation 2%), Et_4_-*anti*-B_18_H_18_ shows an irregular distribution (mean 1.2%; standard deviation 1%), with nearly 60% of cases yielding efficiencies ∼ 0%, i.e., no lasing. Thus, by incorporating the uncertainty in the GMM-NEA spectra, we rationalize with high confidence why this compound does not lase.

As a final assessment of the extent to which excited-state absorption mitigates laser emission from *anti*-B_18_H_22_ and Et_4_-*anti*-B_18_H_18_, we repeated the simulations but, this time, neglecting the excited-state absorption component by setting its cross-section, σ_esa_, to zero. Under these conditions, the laser efficiency of Et_4_-*anti*-B_18_H_18_ increases from ∼ 3% in the presence of excited-state absorption to a remarkable ∼ 58% in its absence, with the laser threshold reduced by more than an order of magnitude, as shown in Figure 7. In addition, in the absence of excited-state absorption, both boranes exhibit very similar laser efficiencies and thresholds, consistent with their comparable spectroscopic parameters. These results unambiguously demonstrate that excited-state absorption is the primary factor responsible for the absence of laser emission from solutions of Et_4_-*anti*-B_18_H_18_, as well as for the unexpectedly low laser efficiency in *anti*-B_18_H_22_.

**Figure 7:**
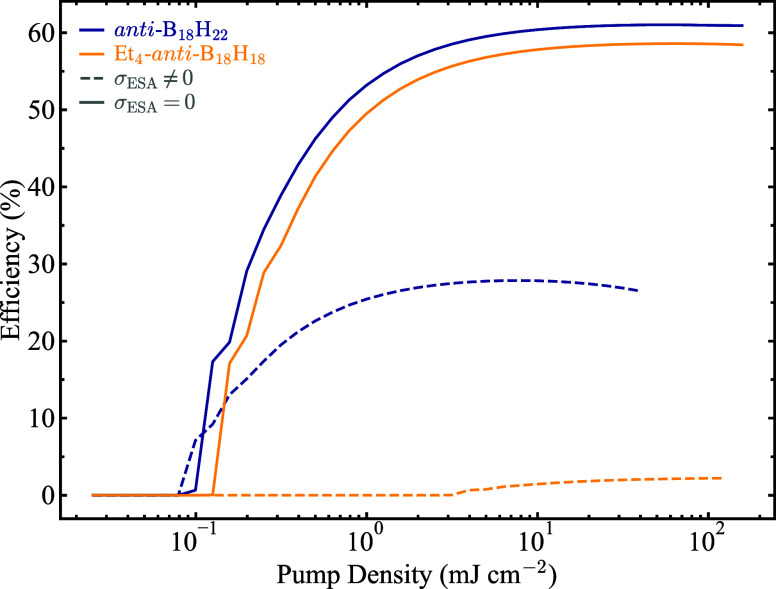
Simulated laser efficiency of solutions of *anti*-B_18_H_22_ (blue lines) and Et_4_-*anti*-B_18_H_18_ (yellow lines) as a function of pump density, in the absence (σ_esa_ = 0, solid lines) and presence (σ_esa_ ≠ 0, dashed lines) of excited-state absorption. The concentration in both cases was set to 10 mM.

Thus, as verified by the above-described analyses, we have successfully validated the proposed methodology for simulating the laser properties of molecular compounds from first principles. While the strategy summarized in Figure 1 performs satisfactorily, several caveats must be considered. First, achieving quantitative agreement between experimental spectra and those computed using QM methods remains notoriously challenging, particularly for medium-to-large size molecules.^83,84^ Both quantitative and qualitative discrepancies can directly impact the simulated laser performance. For example, although the QM calculations in this work are highly accurate and show excellent agreement with the experimental spectra (as demonstrated in Figure 4), the calculated cross-section at the main absorption and emission band peaks still overestimate the experimental ones by a factor of ∼ 2.4. This discrepancy is also evident in the decay rates, with the computed value for *anti*-B_18_H_22_ being γ_r_ ≈ 2.1 × 10^8^ s^–1^, compared to the experimental value γ_r_ ≈ 8.7 × 10^7^ s^–1^. Figure S3 in the Supporting Information shows the experimentally measured laser efficiency as a function of pump density for solutions of *anti*-B_18_H_22_ (green dots), and compare it with simulated values obtained using the GMM-NEA spectra and computed decay rate from eqs 1–6 (blue line), as well as those calculated by rescaling the GMM-NEA results by a factor 1/2.4 (yellow line). Notably, the laser calculations using the as-obtained GMM-NEA spectra predict a laser threshold nearly an order of magnitude lower than the experimental one. In contrast, the simulations with the rescaled GMM-NEA spectra yield an almost perfect agreement with the experimental data, both in terms of laser threshold and efficiency. The quantitative discrepancies between experiment and QM simulations can be attributed to limitations in the methodological approach. In particular, the truncation of the valence orbitals carried out in the selection of the active space of the CASPT2 calculations, necessary to make the computations feasible, affect higher energy states more strongly. Moreover, anharmonicities at the ground- and excited-state minima, as well as solvent effects, may introduce uncertainty in the determination of transition energies and oscillator strengths between electronic excited states.

A second caveat affects the laser simulations. As currently formulated, the model implicitly assumes that the lasing molecules do not interact with one another, irrespective of the concentration. However, under experimental conditions, intermolecular interactions become significant at high concentrations, potentially altering the photophysical properties of the compounds. These changes may include spectral distortions such as band shifts, band narrowing, or the emergence/disappearance of spectral features. In addition, fluorescence quantum yield and associated decay rates can be either enhanced or suppressed depending on the nature of molecular aggregation −specifically, whether the molecules form J- or H-aggregates.^85^ To account for these effects in the laser simulations, one would first need to perform molecular dynamics simulations to determine whether the molecule under study has a tendency to aggregate, and, if so, at what concentration. If aggregation occurs, the GMM-NEA spectra would then need to be recalculated accounting for the aggregated clusters, a task that may be computationally prohibitive. Regarding the laser simulations, we have so far assumed unit photoluminescence quantum yields for both boranes, based on previous knowledge. However, this information may not always be available. The quantum yield ϕ depends on the radiative γ_r_ and nonradiative γ_nr_ decay rates as ϕ = γ_r_/(γ_r_ + γ_nr_). The former can be readily computed with eq 6. Regarding the latter, a first estimation can be obtained by using a static approach referring to Fermi’s Golden Rule for internal conversion and computing the nonadiabatic vibronic coupling.^71^ A more accurate approach, although with a much higher computational cost, would be to perform nonadiabatic molecular dynamics.^70^ In any case, Figure S4 in the Supporting Information displays the simulated laser efficiency of *anti*-B_18_H_22_ as a function of pump density for decreasing values of the quantum yield. This plot reveals that lowering the quantum yield leads to an increase in the laser threshold, while the maximum laser efficiency decreases only moderately.

## Conclusions

In this work, we have presented and thoroughly described a numerical framework for simulating the laser properties −peak wavelength, threshold, and efficiency– of molecular compounds from first principles. It comprises three different components: (i) a block for QM calculations involving geometry optimizations and vertical transition energies calculations for NEA-sampled geometries; (ii) a block for the reconstruction of the NEA electronic spectra employing probabilistic ML (specifically, GMM-NEA); and (iii) a laser simulation block that uses the GMM-NEA spectra as input to model the laser behavior. To enable this, we have extended the GMM-NEA methodology introduced in our previous work by deriving appropriate expressions for both the differential decay rate spectrum (i.e., spontaneous emission) and the stimulated emission cross-section. Additionally, we have developed and tested a spectrally resolved spatiotemporal rate equation model to simulate laser properties based on the GMM-NEA spectra. We have validated the proposed framework and methodologies using two boron hydrides, *anti*-B_18_H_22_ and its alkylated derivative Et_4_-*anti*-B_18_H_18_. Our numerical methodology, which incorporates high-level multireference multiconfigurational calculations via the CASSCF/MS-CASPT2 method, shows sufficient agreement with experimental results to clearly rationalize the lasing properties of the considered molecules. This includes the GMM-NEA reconstructions of the ground- and excited-state absorption spectra and the stimulated emission cross sections, as well as the simulated laser performance. In fact, the latter unambiguously confirms that excited-state absorption is the key factor responsible for the lack of laser emission from solutions of Et_4_-*anti*-B_18_H_18_. Taken together, these results and tests demonstrate the predictive power and versatility of the proposed methodology. As with any numerical approach, special attention must be paid to the limitations and assumptions inherent to the methodologies in the framework. Discrepancies between the experimental photophysical properties and those predicted by the combination of QM computations and GMM-NEA reconstructions can lead to under- or overestimations of the laser performance. Moreover, the assumption that molecular compounds do not interact with each other may add uncertainty to the predictions, particularly at high concentrations where aggregation effects can be significant. The proposed framework is general and modular, as it can be adapted and integrated with methodologies alternative to those used in this contribution. For instance, multiconfiguration methods such as CASSCF can be employed for geometry optimization; the NEA sampling could be obtained from integral path techniques or molecular dynamics simulations to account for solvent effects; and semiclassical laser theories could be adapted to simulate nanophotonic devices based on molecular compounds, among other possibilities. By enabling *in-silico* screening of potential compounds, our strategy opens new possibilities for the rational design of laser materials and offers a robust framework for interpreting experimental data in complex photonic systems.

## Supplementary Material



## Notes

Derivation and implementation of the GMM-NEA expressions for spontaneous and stimulated emission; GMM model selection and optimization; laser rate equations for GMM-NEA spectra. It includes figures with the active space orbitals, GMM-NEA convergence, as well as additional results on the laser efficiency simulations (PDF)
