# Supplementary material for: Predicting Molecular Laser Properties from First-Principles Using Machine Learning-Based Nuclear Ensemble Approach Spectra
Source: J Chem Theory Comput. 2026 Apr 14;22(8):4037–48. doi: 10.1021/acs.jctc.5c01866 (PMC13130866; doi:10.1021/acs.jctc.5c01866)
Supplement: Supplementary file 1 [file ct5c01866_si_001.pdf]

Supporting Information

Predicting Molecular Laser Properties from First Principles using Machine Learning-based Nuclear Ensemble Approach Spectra

Luis Cerdán,[*,†] Antonio Francés-Monerris,[‡] Michael G. S. Londesborough,[¶] and Daniel Roca-Sanjuán[‡]

†*Instituto de Química Física Blas Cabrera (IQF-CSIC), Consejo Superior de Investigaciones Científicas, 28006 Madrid, Spain*

‡*Institut de Ciència Molecular, Universitat de València, P.O. Box 22085, València 46071, Spain*

¶*Institute of Inorganic Chemistry of the Czech Academy of Sciences, Husinec-Řež 250 68, Czech Republic*

E-mail: l.cerdan@csic.es

GMM-NEA for spontaneous and stimulated emission

Within the framework of time-dependent perturbation theory, and under the electric dipole and Born-Oppenheimer approximations, the spontaneous emission spectrum –expressed as the differential radiative decay rate, $\Gamma_{\rm r}(E)$– can be computed using a Monte Carlo nuclear ensemble sampling approach as follows:[1]

$$\Gamma_{\rm r}(E) = \frac{n_r F(n_r)}{3\pi\hbar c^3 \varepsilon_0} \frac{1}{N_g} \sum_{j=1}^{N_g} \Delta E_j^3 \, M_j^2 g_L(E), \tag{S1}$$

where E is the photon energy, c is the speed of light in vacuum, $\hbar$ is the reduced Planck constant, ϵ_0 is the vacuum permittivity, and N_g denotes the number of sampled molecular geometries. For each sampled geometry, M_j and ΔE_j represent the modulus of the dipole moment and the vertical energy for the transition, respectively, from the metastable excited state to the ground state. The transition line-shape $g_L(E)$ is modeled by a normalized Lorentzian $g_L(E) = \delta_L/(2\pi)/((E-\Delta E_j)^2 + (\delta_L/2)^2)$, where δ_L is the full-width half-maximum. Notice that we have expressed equation S1 in terms of M_j instead of oscillator strengths f_j exploiting the relation $M_j^2 = 3\hbar^2 e^2 f_j / 2m\Delta E_j$. It is better to work with M_j instead of f_j to avoid the inherent skewness in the latter, which hinders the adequate fit of GMMs.[2]

Using Equation (S1) and the relationship between the Einstein coefficients,[3–5] we find that the stimulated emission cross-section reads:

$$\sigma_{\rm se}(E) = \frac{\pi F(n_r)}{3\hbar c \varepsilon_0 n_r} \frac{1}{N_g} \sum_{j=1}^{N_g} \Delta E_j \, M_j^2 g_L(E). \tag{S2}$$

To reformulate Equations (S1) and (S2) in terms of GMM parameters, we follow the procedure described in ref. 2. Briefly, Equation (S1) is rewritten as the Lebesgue integral:

$$\Gamma_{\rm r}(E) = \frac{n_r F(n_r)}{3\pi\hbar c^3 \varepsilon_0} \iint_{-\infty}^{+\infty} \Delta E^3 \, M^2 \mathcal{P}(\Delta E, M) g_L(E) d\Delta E dM, \tag{S3}$$

where $\mathcal{P}(\Delta E, M)$ is the joint probability density function (PDF) describing the likelihood of sampling a particular pair $\{\Delta E, M\}$. We can formally describe this PDF as a GMM –a sum of K bivariate normal distributions– along with an additional term accounting for configurations with

null dipole moments, as follows:

$$\mathcal{P}(\Delta E, M) = \sum_{k=1}^{K} \pi_k \phi(\Delta E, M; \boldsymbol{\mu}_k, \boldsymbol{\Sigma}_k) + \Theta_0 \delta(M), \tag{S4}$$

where Θ_0 is the proportion of NEA geometries with $M = 0$ (i.e., forbidden transition), $\delta(\cdot)$ is the Dirac delta distribution, π_k are the weights of the mixtures, and $\phi(\Delta E, M; \boldsymbol{\mu}_k, \boldsymbol{\Sigma}_k)$ are bivariate normal distributions with vector of means $\boldsymbol{\mu}_k = (\mu_{k,1}, \mu_{k,2})$ and covariance matrix $\boldsymbol{\Sigma}_k = (\sigma^2_{k,1}, \rho_k \sigma_{k,1} \sigma_{k,2}; \rho_k \sigma_{k,1} \sigma_{k,2}, \sigma^2_{k,2})$, where $\sigma^2_{k,1}$ and $\sigma^2_{k,2}$ are the variances of the mixture covariates and ρ_k is the correlation coefficient. The subscripts 1 and 2 refer, respectively, to the corresponding variable ΔE and M. Equation (S4) must satisfy the condition $\sum_k \pi_k = 1 - \Theta_0$ to describe a proper joint PDF. Finally, by substituting Equation (S4) into Equation (S3), using the identity $\int \delta(M) dM = 0$, taking the limit where $\delta_L \to 0$ (noting that $\lim_{\delta_L \to 0} g_L(E) = \delta(E - \Delta E)$), and exploiting the properties of the normal distribution,[2] we arrive to the GMM-NEA expression for the differential decay rate given in Equation (4) of the main text. Finally, Equation (S2) is reformulated following the same procedure, yielding the GMM-NEA expression for the stimulated emission cross-section, as given in Equation (5) of the main text.

The statistical uncertainty (confidence intervals, CI) associated with each GMM-NEA spectrum is estimated using a bootstrap procedure, as thoroughly described in our previous work.[2] In short, B subsets of randomly sampled $\{\Delta E, M\}$ pairs are used to fit B different GMMs, each resulting in a bootstrapped spectrum. The lower and upper CI of the reconstructed spectrum are given, respectively, by the 2.5% and 97.5% quantile of the resulting distribution of the B bootstrapped spectra. A narrow CI in the spectrum suggests that the model is not sensitive to the sample selection.

GMM model selection and optimization

A key aspect of ML models in general is *model selection* or which is the best hyperparameter set in terms of generalization and accuracy of the predictions. For the particular case of GMMs, the hyperparameters are the number of components K to include in the mixture, and which constraints

$\mathcal{M}$ to apply to the covariance matrices (spherical, diagonal, or ellipsoidal). Among the many model selection procedures in the context of GMMs,[6] the most common one consists of maximizing the Bayesian Information Criterion (BIC), which is given by:

$$\mathrm{BIC}_{\mathcal{M},K} = -2\ell_{\mathcal{M},K}(\mathbf{x}|\hat{\mathbf{\Psi}}) + \nu \log(n), \tag{S5}$$

where $\ell_{\mathcal{M},K}(\mathbf{x}|\hat{\mathbf{\Psi}})$ is the log-likelihood of the observations $\mathbf{x}$ for a GMM of K components and covariance-matrix structure $\mathcal{M}$ with estimated parameters $\hat{\mathbf{\Psi}}$, n is the sample size, and ν is the number of estimated parameters. Thus, the pair $\{\mathcal{M}, K\}$ maximizing $\mathrm{BIC}_{\mathcal{M},K}$ is selected. The BIC, like other model selection criteria, looks for a compromise between precision (small log-likelihood) and model complexity/simplicity (small number of parameters). The term $\nu \log(n)$ in eq. (S5) acts as a conservative regularization term that penalizes models which are too complex and thus avoids overfitting. This means that even when a more complex GMM could be needed to exactly model the true distribution (if it exists), the BIC could suggest a simpler GMM. In the NEA context, the spectra generated with GMMs could be slightly smoother than the true one.

Alternatively, one could perform a GMM model selection using resampling techniques like leave-n-out or k-fold cross-validation (CV).[6,7] Nevertheless, there is a number of reasons that suggest that using model selection based on information criteria, like BIC, is more adequate that those based on resampling: CV is known to perform poorly or become unstable for small datasets, such as ours;[8] CV requires repeated refitting, while the BIC is computed from a single fit, making it far more efficient; finally, it has been shown that when a GMM is used to estimate a density, as in our case, the density estimate obtained through BIC-based selection of the number of components is consistent.[6,9] Incidentally, the BIC is asymptotically equivalent to leave-η-out CV when $\eta = n(1 - 1/(\log(n) - 1))$.[10] Thus, although we do not explicitly perform held-out validation, our BIC-based selection implicitly corresponds to a theoretically justified form of CV. For all these reasons, the BIC-based approach we use provides a robust guarantee of model quality and generalization in the context of a GMM used as a density estimator.

Laser rate equations for GMM-NEA spectra

Given the laser material and configuration selected for this study, we employed a rate equations model to simulate the laser properties. Specifically, we implemented a spectrally resolved, spatio-temporal set of rate equations for the forward- and backpropagating laser fluxes, $I^{\pm} = I^{\pm}(x, t, \lambda)$, and the population densities of the different levels in Figure 2b of the main text, $N_i = N_i(x, t)$ ($i = 0 \ldots 4$), based on a model successfully applied in previous studies of similar systems.[11–13] Under these conditions, the propagation equations for the photon fluxes are given by:

$$\frac{n_r}{c}\frac{\partial I^{\pm}}{\partial t} \pm \frac{\partial I^{\pm}}{\partial x} = \frac{\Omega\Gamma_{\mathrm{r}}}{4\pi\hbar}N_2 + (N_2 - N_1)\sigma_{\mathrm{se}}I^{\pm} - (N_2 - N_4)\sigma_{\mathrm{esa}}I^{\pm} - (N_0 - N_3)\sigma_{\mathrm{gsa}}I^{\pm}, \tag{S6}$$

where $I^{\pm}$ and N_i have, respectively, units of photons cm^{-2} s^{-1} nm^{-1} and cm^{-3}, $\sigma_i = \sigma_i(\lambda)$ ($i = \mathrm{se}, \mathrm{gsa}, \mathrm{esa}$) are cross-sections in units of nm^2, and $\Omega = wd/(4L^2)$ denotes the solid angle subtended by the excited region. The first term in the right-hand-side of Equation (S6) accounts for the spontaneous emission seed that initiates the laser emission, while the remaining terms correspond to stimulated emission, excited-state absorption, and ground-state absorption, respectively. This equation is complemented by the boundary conditions $I^{+}(0, t, \lambda) = R_1 I^{-}(0, t, \lambda)$ and $I^{-}(L, t, \lambda) = R_2 I^{+}(L, t, \lambda)$, which account for mirror reflections (cavity feedback). It is important to note that Equation (S6) assumes a one-dimensional amplifier, with a homogeneous excitation region in the yz-plane and rectangular, top-hat-shaped propagating fluxes. Additionally, while each wavelength is treated independently, all wavelengths share the same population densities.

The dynamics of the population densities of the different levels satisfies:

$$\frac{dN_4}{dt} =(N_2 - N_4)\left[\sigma_{\mathrm{esa,p}}I_{\mathrm{p}} + \int \sigma_{\mathrm{esa}}(I^{+} + I^{-})d\lambda\right] - N_4\gamma_{\mathrm{vr}}, \tag{S7}$$

$$\frac{dN_3}{dt} =(N_0 - N_3)\left[\sigma_{\mathrm{gsa,p}}I_{\mathrm{p}} + \int \sigma_{\mathrm{gsa}}(I^{+} + I^{-})d\lambda\right] - N_3\gamma_{\mathrm{vr}}, \tag{S8}$$

$$\begin{aligned}\frac{dN_2}{dt} =&(N_3 + N_4)\gamma_{\mathrm{vr}} - N_2(\gamma_{\mathrm{r}} + \gamma_{\mathrm{nr}}) - (N_2 - N_1)\int \sigma_{\mathrm{se}}(I^{+} + I^{-})d\lambda - \\ &- (N_2 - N_4)\left[\sigma_{\mathrm{esa,p}}I_{\mathrm{p}} + \int \sigma_{\mathrm{esa}}(I^{+} + I^{-})d\lambda\right],\end{aligned} \tag{S9}$$

$$\frac{dN_1}{dt} =N_2(\gamma_{\mathrm{r}} + \gamma_{\mathrm{nr}}) - N_1\gamma_{\mathrm{vr}} + (N_2 - N_1)\int \sigma_{\mathrm{se}}(I^{+} + I^{-})d\lambda, \tag{S10}$$

$$N_0 = N_\mathrm{D} - \sum_{i=1}^{4} N_i, \tag{S11}$$

where γ_r, γ_nr, and γ_vr are radiative, non-radiative, and vibrational relaxation decay rates, respectively. The terms $\sigma_\mathrm{gsa,p}$ and $\sigma_\mathrm{esa,p}$ represent the ground- and excited-state absorption cross-sections evaluated at λ_p, and $I_\mathrm{p} = I_\mathrm{p}(t)$ is the time-dependent pump rate, which follows the Gaussian pulse profile:[13]

$$I_\mathrm{p} = \frac{\lambda_p}{hc} \frac{2\sqrt{\ln 2}}{\sqrt{\pi}} \frac{E_\mathrm{p}}{wL\tau_p} \exp\left(-4 \ln 2 \frac{t^2}{\tau_\mathrm{p}^2}\right) \tag{S12}$$

with h the Planck's constant, and E_p and τ_p the pump pulse energy and duration, respectively. We solved the resulting system of equations, along with the appropriate boundary conditions, using the numerical integration method described in ref. 12.

Supplementary Figures

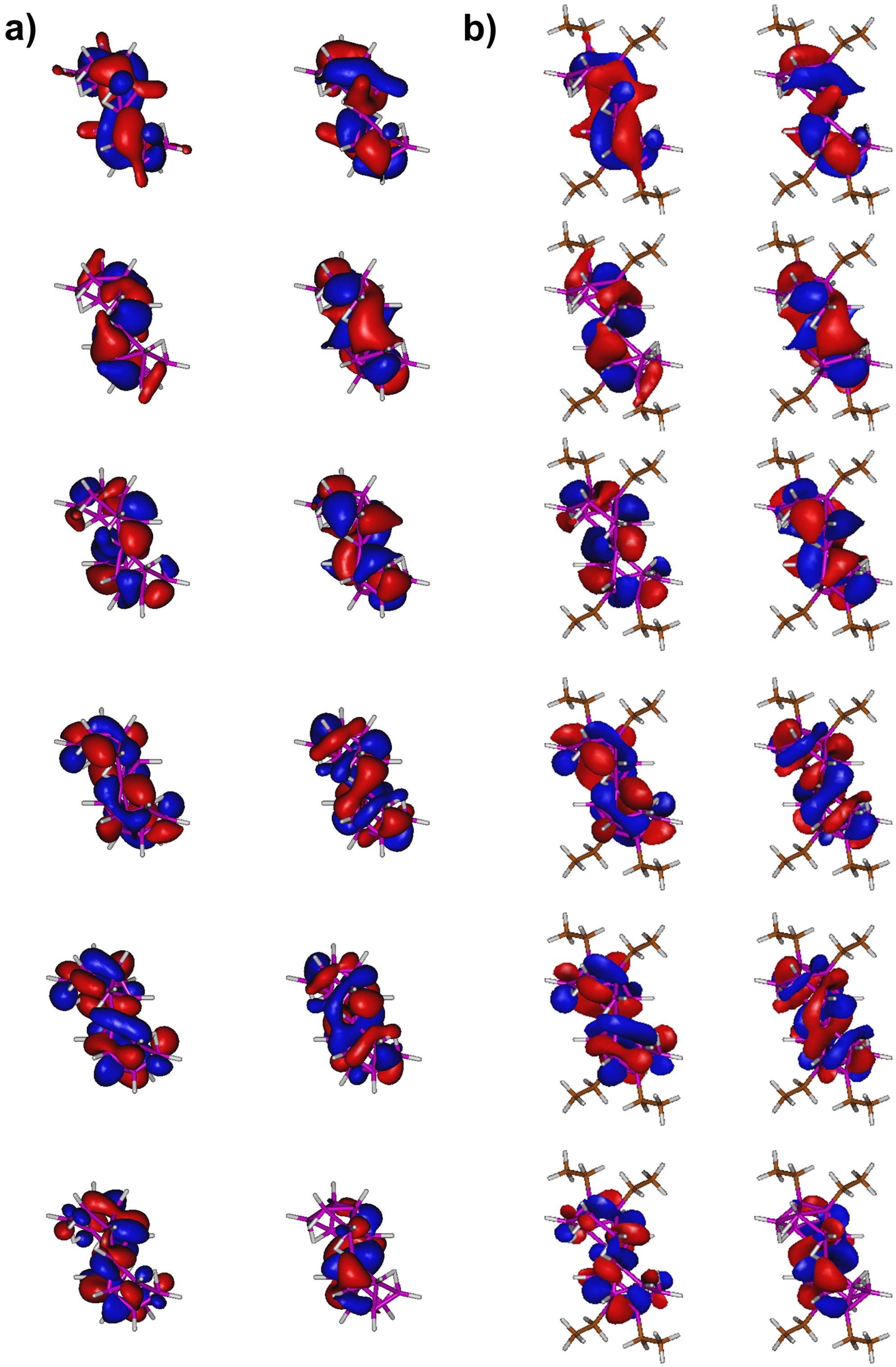

Figure S1: Complete-active-space (CAS) of 12 electrons distributed into 12 orbitals used in this work for (a) *anti*-$B_{18}H_{22}$ and (b) Et_4-*anti*-$B_{18}H_{18}$.

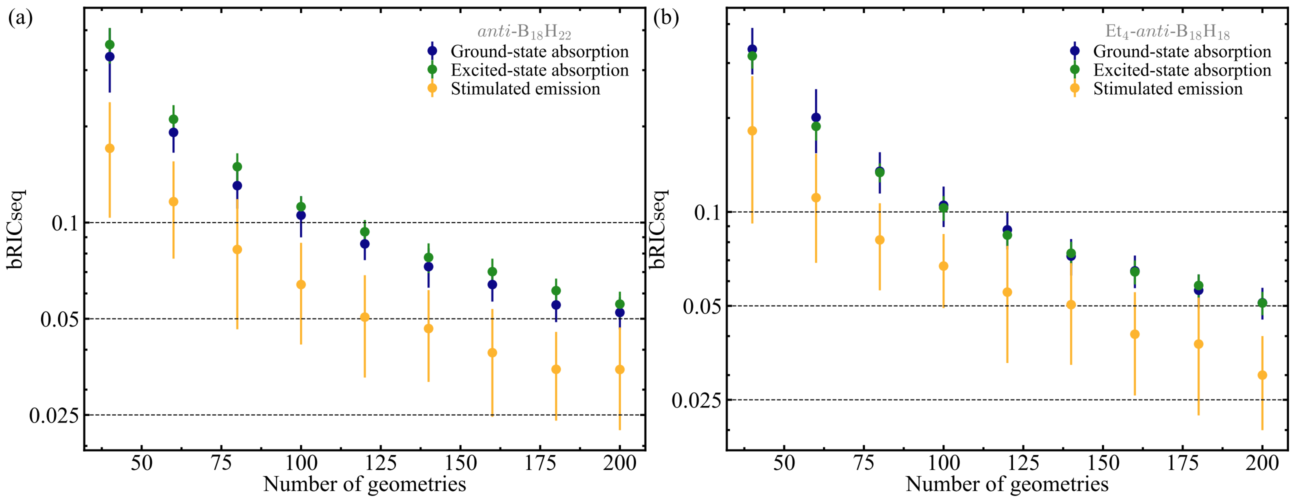

Figure S2: Sequential band-wise relative integral change ($bRIC_{\mathrm{seq}}$), computed with a batch size of 20, for ground-state absorption (blue dots), excited-state absorption (green dots), and stimulated emission (yellow dots) for (a) *anti*-$B_{18}H_{22}$ and (b) Et_4-*anti*-$B_{18}H_{18}$. Markers and error bars represent the average and standard deviation over 40 independent sequential draws. The $bRIC_{seq}$ metric is defined as $bRIC_{seq} \doteq N_s^{-1} \sum_{n=1}^{N_s} \left[\int |\sigma_{R,n}^{old}(E) - \sigma_{R,n}^{new}(E)| dE / \int \sigma_{R,n}^{old}(E) dE \right]$, where $\sigma_{R,n}^{old}(E)$ and $\sigma_{R,n}^{new}(E)$ are, respectively, the reconstructed electronic spectra for band n before and after adding a new batch of data.

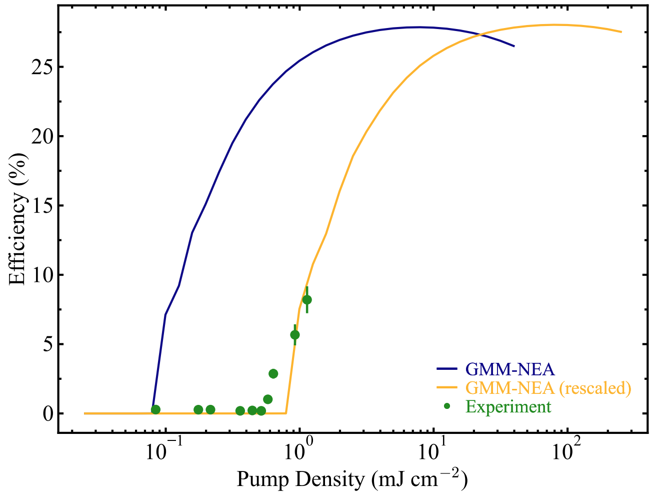

Figure S3: Laser efficiency as a function of pump density for 25 mM solutions of *anti*-$B_{18}H_{22}$ as obtained from: Experimental data from ref. 14 (green dots); laser simulations using the GMM-NEA spectra as calculated with Equations (1)-(6) of the main text, assuming a quantum yield of 1 (blue line); laser simulations using GMM-NEA spectra and the decay rate rescaled by a factor 1/2.4, and assuming the experimentally measured quantum yield of 0.86 (yellow line).

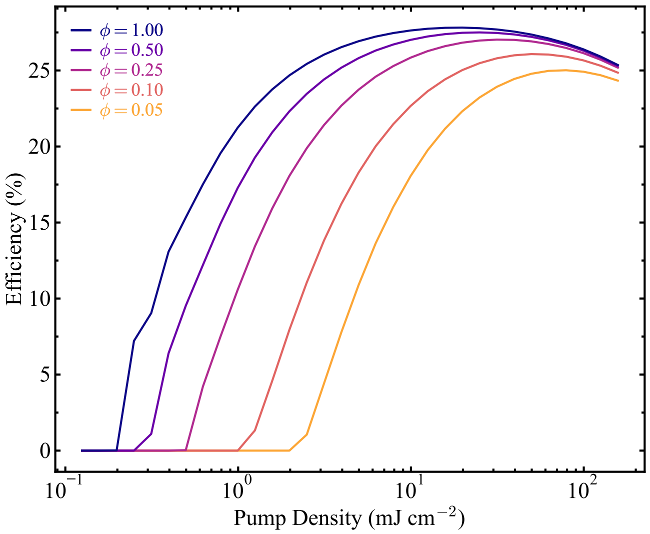

Figure S4: Simulated laser efficiency as a function of pump density for 10 mM solutions of *anti*-$B_{18}H_{22}$, evaluated for decreasing values of the photoluminescence quantum yield ϕ.

References

(1) Crespo-Otero, R.; Barbatti, M. Spectrum simulation and decomposition with nuclear ensemble: formal derivation and application to benzene, furan and 2-phenylfuran. *Theor. Chem. Acc.* **2012**, *131*, 1237.

(2) Cerdán, L.; Roca-Sanjuán, D. Reconstruction of Nuclear Ensemble Approach Electronic Spectra Using Probabilistic Machine Learning. *J. Chem. Theory Comput.* **2022**, *18*, 3052–3064.

(3) Siegman, A. E. *Lasers*; University Science Books, 1986.

(4) Silfvast, W. *Laser Fundamentals*; Cambridge University Press, 2008.

(5) Hilborn, R. C. Einstein coefficients, cross sections, f values, dipole moments, and all that. *Am. J. Phys.* **1982**, *50*, 982–986.

(6) McLachlan, G. J.; Rathnayake, S. On the number of components in a Gaussian mixture model. *WIRES Data Min. Knowl.* **2014**, *4*, 341–355.

(7) Smyth, P. Model selection for probabilistic clustering using cross-validated likelihood. *Stat. Comput.* **2000**, *10*, 63–72.

(8) Varoquaux, G. Cross-validation failure: Small sample sizes lead to large error bars. *NeuroImage* **2018**, *180*, 68–77.

(9) Roeder, K.; Wasserman, L. Practical Bayesian Density Estimation Using Mixtures of Normals. *J. Am. Stat. Assoc.* **1997**, *92*, 894–902.

(10) Shao, J. An asymptotic theory for linear model selection. *Stat. Sin.* **1997**, *7*, 221–242.

(11) Ganiel, U.; Hardy, A.; Neumann, G.; Treves, D. Amplified spontaneous emission and signal amplification in dye-laser systems. *IEEE J. Quantum Electron.* **1975**, *11*, 881–892.

(12) Weigand, R.; Guerra, J. M. Study of acid-base dye laser systems. *Opt. Quantum Electron.* **1995**, *27*, 1027–1051.

(13) Cerdan, L.; Costela, A.; Garcia-Moreno, I.; Martin, V.; Perez-Ojeda, M. E. Laser Efficiency Enhancement Due to Non-Resonant Feedback in Dye-Doped Hybrid Materials: Theoretical Insights and Experiment. *IEEE J. Quantum Electron.* **2011**, *47*, 907–919.

(14) Cerdán, L.; Braborec, J.; Garcia-Moreno, I.; Costela, A.; Londesborough, M. G. S. A borane laser. *Nat. Commun.* **2015**, *6*, 1–7.
